# Cardiovascular effects of resveratrol and atorvastatin treatments in an H_2_O_2_-induced stress model

**DOI:** 10.3892/etm.2014.1956

**Published:** 2014-09-11

**Authors:** BURAK CEM SONER, AYŞE SAIDE ŞAHIN

**Affiliations:** Department of Medical Pharmacology, Meram Medical Faculty, Necmettin Erbakan University, Konya 42080, Turkey

**Keywords:** resveratrol, atorvastatin, myocardium, hydrogen peroxide, endothelium

## Abstract

Oxidative stress has been implicated in the pathophysiology of several types of cardiovascular disease (CVD). Statins are widely used to inhibit the progression of atherosclerosis and reduce the incidence of CVD. Certain over-the-counter products, including resveratrol, show similar effects to statins and may thus be used in conjunction with statins for the treatment of the majority of patients with CVD. The aim of the present study was to evaluate the effects of atorvastatin, resveratrol and resveratrol + atorvastatin (R+A) pretreatment on myocardial contractions and vascular endothelial functions in the presence of H_2_O_2_ as an experimental model of oxidative stress in rats. Four groups were established and referred to as the control, atorvastatin, resveratrol and R+A groups. Atorvastatin (40 mg/kg, per oral) and/or resveratrol (30 mg/kg, intraperitoneal) treatments were administered for 14 days. On the 15th day, the thoracic aortas and hearts of the rats were dissected and placed into isolated organ baths. Vascular responses to cumulative doses of H_2_O_2_ (1×10^−8^–1×10^−4^ M H_2_O_2_) with and without N (G)-nitro-L-arginine methyl ester (L-NAME) incubation were measured. In addition, myocardial electrical stimulation (ES) responses to various H_2_O_2_ concentrations (1×10^−7^–1×10^−5^ M H_2_O_2_) were evaluated. In the control and atorvastatin groups, H_2_O_2_ application caused a significant dose-dependent decrease in the ES-induced contractions in the myocardial tissue of rats. In the resveratrol and R+A groups, H_2_O_2_ application did not significantly affect myocardial contraction at any dose. In all groups, incubation with L-NAME caused a significant augmentation in the H_2_O_2_ response, revealing that this effect was mediated via the vascular endothelium. In conclusion, pretreatment with R+A for CVD appears to be superior to pretreatment with either agent alone.

## Introduction

Oxidative stress has been implicated in the pathophysiology of several types of cardiovascular disease (CVD), including ischemic stroke, myocardial ischemia, myocardial stunning, ischemia-reperfusion injury, hypertension and atherosclerosis. It is also considered to play a role in the progression of atherosclerosis (1–4). Previous studies have demonstrated that the majority of patients with CVD are likely to have chronic oxidative stress and that this is associated with their diagnosed disease state (5–7). H_2_O_2_ is an important byproduct of oxidative metabolism and is a major contributor to oxidative stress-induced functional and metabolic dysfunction.

The β-hydroxy-β-methylglutaryl coenzyme A (HMG-CoA) reductase inhibitors (statins) are widely used to inhibit the progression of atherosclerosis and reduce the incidence of CVD. As well as their cholesterol-lowering effects, statins improve endothelial function in normocholesterolemia (8,9). Certain over-the-counter (OTC) products, including resveratrol, also exhibit similar effects to statins. Resveratrol (trans-3,5,4-trihydroxystilbene) is a polyphenol (phytoalexin) that naturally occurs in red wine and in a variety of therapeutic plants. *In vitro* experiments have revealed that the cardiovascular protective effects of resveratrol may occur through a number of mechanisms. Resveratrol inhibits the proliferation of smooth muscle cells, platelet aggregation and the oxidation of low-density lipoprotein cholesterol, and reduces the synthesis of lipids and eicosanoids, which promote inflammation and atherosclerosis (10). These multiple protective effects of resveratrol increase its demand as an OTC product, even for those undergoing treatment with statins.

A number of studies have demonstrated the aggravating effects of statins on oxidative stress in organisms (11,12). Such aggravating effects of statins on the myocardium have already been shown (13,14,15,16). The aim of the present study was to evaluate the effects of atorvastatin, resveratrol and resveratrol + atorvastatin (R+A) pretreatment on myocardial contractions and endothelial function in the presence of H_2_O_2_ as an experimental model of oxidative stress in rats.

## Materials and methods

### Animals and experimental procedure

A total of 28 male Wistar albino rats, aged 8 weeks and weighing 260–280 g, obtained from the Animal Care Facility of Meram Medical Faculty (Konya, Turkey) were used in the present study. Animals were housed identically in cages in an air-conditioned room under a 12-h light/dark cycle. Temperature and relative humidity were controlled within the limits of 21±2°C and 55±15%, respectively. All animals were acclimated for ≥7 days prior to the onset of the study. A standard diet and tap water were provided *ad libitum*. The experimental procedures were approved by the Animal Ethics Committee of the Meram School of Medicine (Konya, Turkey). All chemicals were purchased from Sigma-Aldrich (St. Louis, MO, USA) and stored according to the manufacturers’ instructions unless otherwise specified. For 14 days, the control group (n=8) received 1.5 ml drinking water by oral gavage and 1 ml 10% v/v dimethyl sulfoxide [DMSO; intraperitoneal (i.p.)], the vehicle for resveratrol. The atorvastatin group (n=6) received 40 mg/kg atorvastatin (Lipitor^®^), which was prepared daily and dissolved in drinking water, by oral gavage and 1 ml 10% v/v DMSO i.p. for the same period of time. The resveratrol group (n=6) was treated with 30 mg/kg i.p. resveratrol and 1.5 ml drinking water by oral gavage for the 14 days, and the R+A group (n=8) was treated with 40 mg/kg atorvastatin by oral gavage and 30 mg/kg i.p. resveratrol. Rats were weighed every five days for adjustments to the dosing schedule and observed every day or as necessary. On day 15, the rats were anesthetized with an i.p. injection of 50 mg/kg body weight sodium pentobarbital. The heart and thoracic aorta were removed from each rat and placed immediately into fresh, oxygenated, ice-cold Krebs-Henseleit solution (KHS) composed of 119 mmol/l NaCl, 4.7 mmol/l KCl, 1.5 mmol/l MgSO_4_, 1.2 mmol/l KH_2_PO_4_, 2.5 mmol/l CaCl_2_, 25 mmol/l NaHCO_3_ and 11 mmol/l glucose.

### Detection of myocardial contraction

Myocardial strips (10–13 mm long and 2–3 mm wide) were prepared from the left ventricle using a previously described method (17). The strips were placed in a 10-ml chamber containing oxygen-enriched (0.5 l/min) KHS at 37°C. One end of the strip was attached to a force transducer (MP30; Biopac Systems, Inc., Santa Barbara, CA, USA) by a thin silk thread and the other end was attached to a hook in the tissue bath. The strips were left for 30 min for stabilization. Following the stabilization period, the maximum contractions to electrical stimulation (ES) were recorded [frequency, 0.5 Hz; duration, 5 msec; and voltage, 30–40 V (20% above threshold)]. The effects of H_2_O_2_ on myocardial contractions were then evaluated at concentrations of 1×10^−7^, 1×10^−6^ and 1×10^−5^ M. Following each dose, the organ bath was washed with KHS and the next dose was applied after 20 min resting time. The same procedure was conducted for all four groups. Contractions are given as a percentage of the initial contractions.

### Detection of thoracic aorta responses

Thoracic aorta rings (2–3 mm wide) were placed into a 10-ml chamber containing oxygen-enriched (0.5 l/min) KHS at 37°C. One end of the strip was attached to a force transducer (MP30; Biopac Systems, Inc.) by a thin silk thread, while the other end was pinned to a hook in the tissue bath. The strips were left for 15 min to spontaneously recover their isometric tension, following which they were gradually stretched to a resting force of 1 × g. The tissues were allowed to equilibrate for 30 min with repeated washing every 10 min with KHS. Following the equilibration period, the thoracic rings were contracted with 80 mM KCl. After the 30-min wash-out period in which the tissues were repeatedly washed every 10 min with KHS, 1×10^−8^, 1×10^−7^, 1×10^−6^, 1×10^−5^ and 1×10^−4^ M H_2_O_2_ were cumulatively added to the organ bath. Once the contractions reached a plateau, the tissues were washed twice every 15 min and incubated with 1×10^−4^ M N (G)-nitro-L-arginine methyl ester (L-NAME), an inhibitor of nitric oxide (NO) formation, for 30 min to evaluate the effect of the vascular endothelium on the H_2_O_2_ results. Following incubation, 1×10^−8^, 1×10^−7^, 1×10^−6^, 1×10^−5^ and 1×10^−4^ M H_2_O_2_ were cumulatively added to the organ bath once more. All results are expressed as a percentage of the previous contraction induced by 80 mM KCl.

### Statistical analysis

Data are expressed as the mean ± standard error of the mean. The statistical significance of differences between the groups was analyzed by one-way analysis of variance or the Student’s t-test. P<0.05 was considered to indicate a statistically significant difference.

## Results

### Intragroup myocardial results

H_2_O_2_ was applied to the organ bath at doses of 1×10^−7^, 1×10^−6^ and 1×10^−5^ M. To observe the effects of increasing doses of H_2_O_2_, an intragroup comparison of the myocardial results was carried out for each group. In the control group, H_2_O_2_ significantly reduced the contractions induced by ES at all doses (1×10^−7^ vs. 1×10^−6^ M and 1×10^−6^ vs. 1×10^−5^ M, P<0.01). The results were 94.16±5.94, 80.35±5.66 and 62.61±8.28% for doses of 1×10^−7^, 1×10^−6^ and 1×10^−5^ M, respectively. In the rats treated with atorvastatin, H_2_O_2_ caused a significant dose-dependent decrease in myocardial contractions (72.09±3.80, 66.59±3.14 and 48.96±8.93% for H_2_O_2_ doses of 1×10^−7^, 1×10^−6^ and 1×10^−5^ M, respectively; 1×10^−5^ vs. 1×10^−7^ M and 1×10^−6^ M, P<0.01). In the resveratrol group, no significant changes in contraction were observed following H_2_O_2_ application at all doses (87.91±2.33, 89.66±14.91 and 79.77±17.33% for H_2_O_2_ doses of 1×10^−7^, 1×10^−6^ and 1×10^−5^ M, respectively; P>0.05). In the R+A group, the contraction results following H_2_O_2_ application were 76.57±1.40, 66.34±5.91 and 66.55±11.10% for 1×10^−7^, 1×10^−6^ and 1×10^−5^ M H_2_O_2_, respectively; H_2_O_2_ application did not significantly decrease the contractions when its concentration was increased.

### Intergroup myocardial results

**Figure 1:**
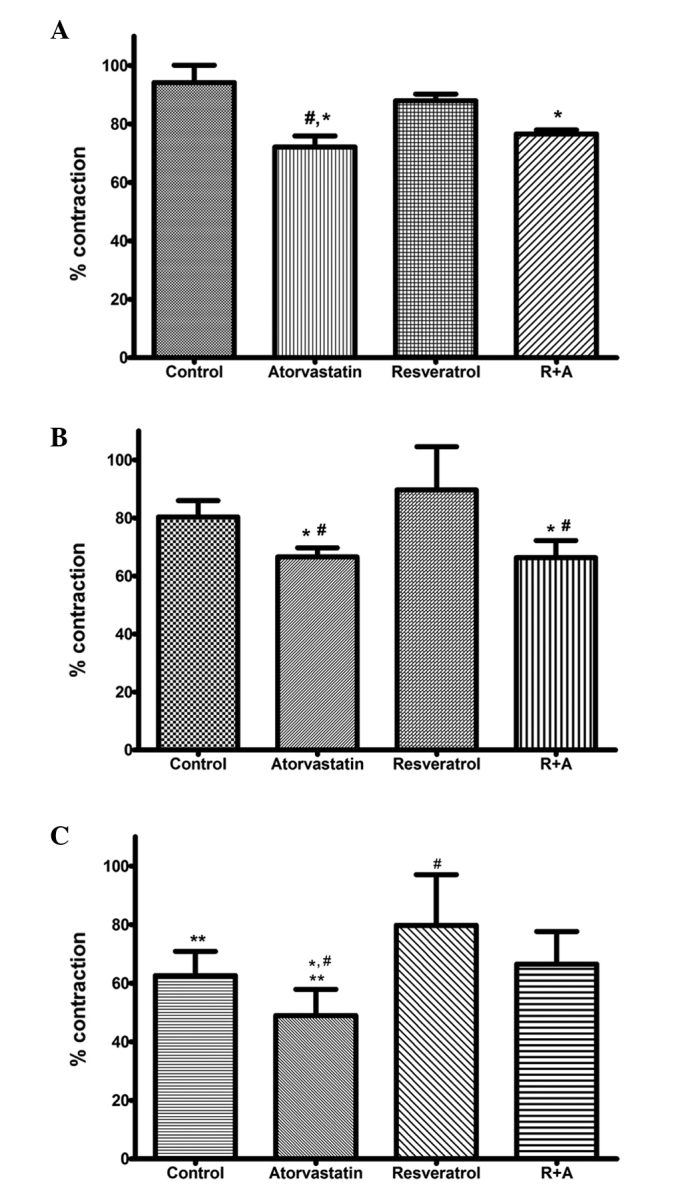
(A) Effects of ES with 1×10^−7^ M H_2_O_2_ on rat myocardium.^*^P<0.01 vs. control group; ^#^P<0.01 vs. resveratrol group. (B) Myocardial effects of ES with 1×10^−6^ M H_2_O_2_. ^*^P<0.05 vs. control group; ^#^P<0.01 vs. resveratrol group. (C) Myocardial effects of ES with 1×10^−5^ M H_2_O_2_. ^*^P<0.05 vs. control group; ^#^P<0.05 vs. R+A group; ^**^P<0.01 vs. resveratrol group. ES, electrical stimulation; R+A, resveratrol + atorvastatin.

Intergroup comparisons of the myocardial results were evaluated for all doses of H_2_O_2_. At 1×10^−7^ M H_2_O_2_ the atorvastatin group showed a significantly lower contraction percentage when compared with the control and resveratrol groups (P<0.01). The R+A group also demonstrated a significant decrease in contraction percentage when compared with the control group (P<0.01). However, no significant difference was observed between the contraction percentages of the resveratrol and control groups (Fig. 1A). Following a 20-min washing period, the organ bath was adjusted to 1×10^−6^ M H_2_O_2_. The myocardial contractions of the atorvastatin and R+A groups were significantly lower than those control group (P<0.05). The resveratrol group tissues showed a significantly higher percentage contraction than those of the atorvastatin and R+A groups (P<0.01). No significant difference was observed between the myocardial contractions in the resveratrol and control groups (Fig. 1B). At the final dose of H_2_O_2_ (1×10^−5^ M), the atorvastatin group exhibited a significant decrease in contraction compared with all the other groups (atorvastatin versus control and R+A groups, P<0.05; atorvastatin versus resveratrol group; P<0.01). The results of the present study demonstrated that resveratrol treatment alone attenuated the decrease in contraction percentage and that this effect was significant compared with all groups at 1×10^−5^ M H_2_O_2_ (P<0.01 vs. atorvastatin and control and P<0.05 vs. R+A). In the R+A group, the contraction percentages were higher than those in the atorvastatin group but significantly lower than those in the resveratrol group (P<0.05) (Fig. 1C).

### Results of thoracic aorta responses

**Figure 2:**
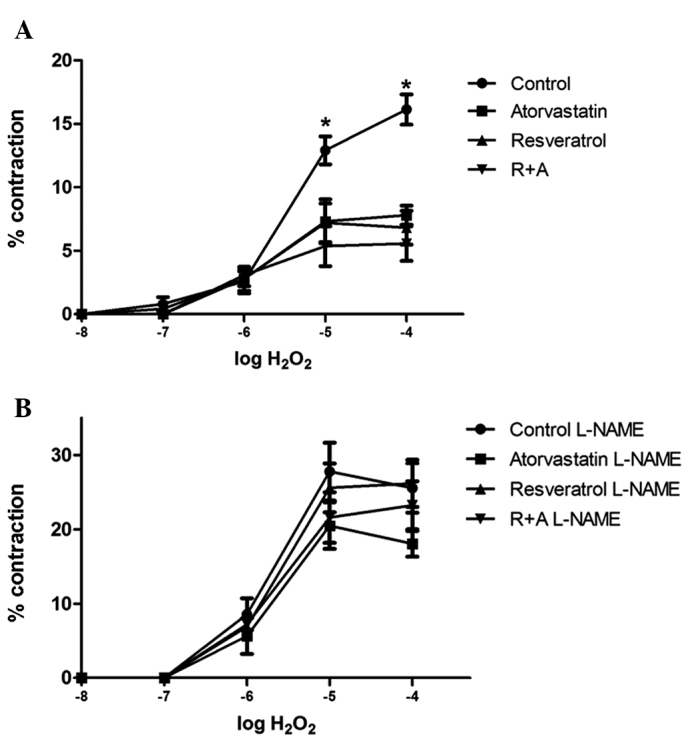
(A) Cumulative contractile response elicited by H_2_O_2_ (1×10^−8^–1×10^−4^ M) in aorta segments. ^*^P<0.05 vs. treatment groups. (B) The same procedure but showing results following 30 min incubation with 1×10^−4^ M L-NAME. R+A, resveratrol + atorvastatin; L-NAME, N (G)-nitro-L-arginine methyl ester.

**Figure 3:**
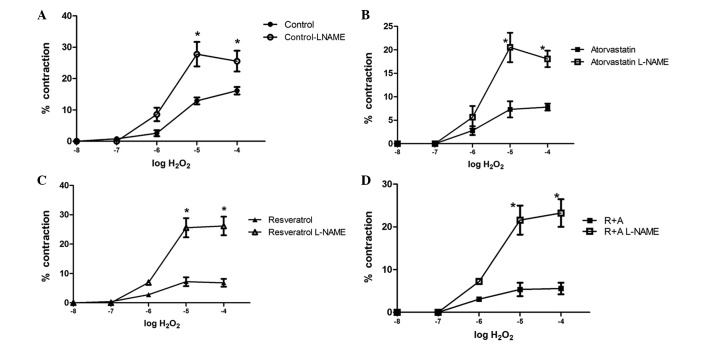
Effect of cumulative concentrations of H_2_O_2_ on basal tone. Comparison between incubation of aortic segments with and without L-NAME in the (A) control, (B) atorvastatin-treated, (C) resveratrol-treated and (D) R+A-treated groups. R+A, resveratrol + atorvastatin; L-NAME, N (G)-nitro-L-arginine methyl ester.

Fig. 2 shows the cumulative dose responses to H_2_O_2_ (between 1×10^−8^ and 1×10^−4^ M) in aortic segments for the control, resveratrol, atorvastatin and R+A groups, with and without 1×10^−4^ M L-NAME incubation. In all groups, H_2_O_2_ caused vasoconstriction and the contraction responses increased in a concentration-dependent manner. The aortic rings reached their maximum contraction at 1×10^−4^ M H_2_O_2_, and the maximum contraction responses were 16.14±1.09, 7.50±0.75, 6.82±1.33 and 5.58±1.37% for the control, atorvastatin, resveratrol and R+A groups, respectively. The H_2_O_2_ responses were significantly lower in the treatment groups than those in the control group at 1×10^−4^ and 1×10^−5^ M H_2_O_2_ (control versus atorvastatin and resveratrol groups, P<0.05; control versus R+A group, P<0.01). When the maximum vasoconstriction responses of the treatment groups were examined, no statistical differences were identified among the resveratrol, atorvastatin and R+A groups (Fig. 2A). Following incubation with 1×10^−4^ M L-NAME for 30 min, the contraction responses of the tissues to H_2_O_2_ significantly increased when compared with their previous responses (Fig. 2B). In all groups, L-NAME incubation significant augmented the H_2_O_2_ response at 1×10^−5^ and 1×10^−4^ M when compared with the response without L-NAME incubation (Fig. 3). The maximum responses were 25.56±3.32, 18.06±1.74, 26.19±3.17 and 23.24±3.24% for the control, atorvastatin, resveratrol and R+A groups, respectively.

The thoracic aorta responses demonstrated that treatment of rats with resveratrol, atorvastatin and R+A resulted in a significantly lower vascular contraction response to H_2_O_2_ at a concentration 1×10^−4^ M when compared with the control group. However, this result was eliminated with 1×10^−4^ M L-NAME incubation.

## Discussion

The cardiac results of the present study revealed that oral administration of 40 mg/kg atorvastatin for 14 days resulted in a more sensitive myocardial response to H_2_O_2_ in rats. Treatment with 30 mg/kg i.p. resveratrol showed a cardioprotective effect against atorvastatin-aggravated and H_2_O_2_-induced contractile dysfunction in the rat myocardium. Furthermore, the study revealed that atorvastatin and resveratrol exhibited a protective effect against H_2_O_2_-induced vasoconstriction and that this protective effect was mediated by NO.

Vasoconstriction induced by cumulative concentrations of H_2_O_2_ was augmented with L-NAME incubation. These results indicated that the vasoconstriction elicited by high concentrations of H_2_O_2_ was negatively modulated by the endothelium. NO exerted a protective effect to counteract the oxidative effect of H_2_O_2_ in the groups without L-NAME incubation. The protective effect of NO on H_2_O_2_ in endothelial monolayer permeability has been previously demonstrated by McQuaid *et al* (18). In their study, it was concluded that, although lower levels of NO may only give a small amount of cytoprotection, the barrier dysfunction in the endothelium caused by H_2_O_2_ may be partially reversed by NO (13). It may be concluded from the results of the present study that the protective effect of resveratrol and/or atorvastatin treatment may be attributed to increased NO production in the vascular endothelium.

Under normal conditions, the H_2_O_2_ concentrations in human plasma, blood and vascular cells are likely to be in the lower micromolar ranges or below. However, in pathological states, including myocardial ischemia and heart failure, it has been demonstrated that H_2_O_2_ concentrations can increase to millimolar levels (19–21). Atorvastatin impairs cholesterol production by inhibiting the synthesis of mevalonate. In addition to cholesterol-lowering effects, statins inhibit the biosynthesis of the major natural antioxidants ubiquinone (ubiphenol) Q10 and glutathione peroxidase (22–24). As a result of this, statins may aggravate oxidative stress in the organism. Such aggravating effects of statins on the myocardium have previously been demonstrated (16). These changes may modulate myocardial contractility. A previous study revealed that an increase in reactive oxygen species (ROS) in the myocardium resulted in ischemia, reperfusion injury and myocardial damage (25).

Studies on the antioxidant effects of statins have been performed using oxidative stress markers (OSMs) in body fluids (26–29). Although OSMs are accepted to reflect the levels of oxidative stress within tissues, Argüelles *et al* (30) demonstrated that OSMs were not correlated with the tissue levels of oxidative stress; furthermore, they suggested that OSMs did not reflect the local oxidative stress status of individual organs. This is consistent with the results of the present study, in which atorvastatin treatment aggravated the H_2_O_2_ response in the myocardial tissue and showed a protective effect on H_2_O_2_-induced vascular contractions. Although the current study did not assess the antioxidant capacity of the myocardium, the diminished myocardial response may be attributed to decreased levels of antioxidant agents in the myocardium, including ubiquinone Q10, whose production is HMG-CoA reductase-dependent (31). The protective effect of resveratrol can be attributed to its inhibitory effect on ROS production in the myocardium (32).

Increased levels of pro-oxidants have been associated with vascular diseases and they are considered to be an important initial step in the development of vascular diseases, including atherosclerosis and hypertension (33). The present study demonstrated that atorvastatin treatment disrupted ES-induced myocardial function in the presence of H_2_O_2_, but that its co-treatment with resveratrol recovered this effect. R+A treatment also exhibited a protective effect on H_2_O_2_-induced vascular responses. From these results, resveratrol appears to be a promising treatment for the improvement of myocardial function in diseases associated with the development of oxidative stress. Resveratrol has been a popular choice in OTC products. Resveratrol is frequently used for the prevention of atherosclerosis; thus, the indications for its administration appear to be similar to those for the administration of statins. The combined treatment of R+A provides a superior treatment for CVD compared with treatment with either agent alone.
